# Both FA- and mPEG-conjugated chitosan nanoparticles for targeted cellular uptake and enhanced tumor tissue distribution

**DOI:** 10.1186/1556-276X-6-563

**Published:** 2011-10-25

**Authors:** Zhenqing Hou, Chuanming Zhan, Qiwei Jiang, Quan Hu, Le Li, Di Chang, Xiangrui Yang, Yixiao Wang, Yang Li, Shefang Ye, Liya Xie, Yunfeng Yi, Qiqing Zhang

**Affiliations:** 1 Research Center of Biomedical Engineering, Material College, Xiamen University, Xiamen 361005, China; 2 First Hospital, Xiamen University, Xiamen, 361003, China; 3 Southeast Hospital, Xiamen University, Zhangzhou, 363000, China; 4 Tianjin Key Laboratory of Biomedical Materials, Tianjin 300192, China

**Keywords:** chitosan, nanoparticles, drug delivery, mitomycin C

## Abstract

Both folic acid (FA)- and methoxypoly(ethylene glycol) (mPEG)-conjugated chitosan nanoparticles (NPs) had been designed for targeted and prolong anticancer drug delivery system. The chitosan NPs were prepared with combination of ionic gelation and chemical cross-linking method, followed by conjugation with both FA and mPEG, respectively. FA-mPEG-NPs were compared with either NPs or mPEG-/FA-NPs in terms of their size, targeting cellular efficiency and tumor tissue distribution. The specificity of the mPEG-FA-NPs targeting cancerous cells was demonstrated by comparative intracellular uptake of NPs and mPEG-/FA-NPs by human adenocarcinoma HeLa cells. Mitomycin C (MMC), as a model drug, was loaded to the mPEG-FA-NPs. Results show that the chitosan NPs presented a narrow-size distribution with an average diameter about 200 nm regardless of the type of functional group. In addition, MMC was easily loaded to the mPEG-FA-NPs with drug-loading content of 9.1%, and the drug releases were biphasic with an initial burst release, followed by a subsequent slower release. Laser confocal scanning imaging proved that both mPEG-FA-NPs and FA-NPs could greatly enhance uptake by HeLa cells. In vivo animal experiments, using a nude mice xenograft model, demonstrated that an increased amount of mPEG-FA-NPs or FA-NPs were accumulated in the tumor tissue relative to the mPEG-NPs or NPs alone. These results suggest that both FA- and mPEG-conjugated chitosan NPs are potentially prolonged drug delivery system for tumor cell-selective targeting treatments.

## Introduction

There is a wealth of literature related to the development of drug delivery carriers for cancer and other diseases. Various drug delivery carriers such as NPs, liposomes, and micelles display significantly improved therapeutic efficacy against different tumors. The nanosized particles can circulate in the bloodstream for longer time and offer unique possibilities to overcome cellular barriers, thus reaching tumor sites more effectively. It has become apparent that, when administered systemically, the biocompatible NPs preferentially accumulate in solid tumors by the enhanced permeability and retention (EPR) effect [1,2], attributed to leaky tumor vessels and lack of the effective lymphatic drainage system. Among the nanosized particles previously reported, the chitosan NPs [3,4] had drawn increasing attention as a drug carrier because of its advantages for biomedical applications such as biocompatibility, biodegradability, and biological activities [5-7]. Besides, the reactive amino groups in the backbone of chitosan make it possible to chemically conjugate various biological molecules such as different ligands and antibodies, which may improve targeting efficiency of the drug to the site of action [8,9].

Berthold et al. [10] initially prepared chitosan particles using sodium sulfate as the precipitation agent. Tian and Groves [11] improved this technique and obtained 600-800-nm chitosan NPs. Ohya et al. [12] used glutaraldehyde as a cross-linking agent to cross-link the free amino groups of chitosan, then emulsified using W/O emulsifier, producing 5-fluorouracil chitosan particles (average particle size, 0.8 ± 0.1 μm). Bodmeier et al. [13] first applied the ionic cross-linking method to prepare chitosan NPs. Tokumitsu et al. [14] reported that it was easy to incorporate drugs in chitosan solution by adding an emulsifier and agitating at high speed to produce 426 ± 28-nm chitosan NPs. Chitosan NPs have been prepared either by a method of emulsion cross-linking with dialdehydes or by a method of ionic gelation with multivalent anions such as tripolyphosphate. Both of the methods have their disadvantages. The former method needs a large amount of organic solvent (consisting of light liquid paraffin and heavy liquid paraffin) to serve as continuous oil phase [15], and the latter have poor mechanical strength because of weak ionic bond formed through an electrostatic attraction between chitosan and sodium triphosphate (STPP) [16]. In this study, an ionic gelation combined with chemical cross-linking method was used to prepare chitosan NPs in order to overcome their disadvantages. However, chitosan NPs used as a drug delivery system must be present in the circulation for enough time to reach to its intended target tissue. Plasma proteins can bind circulating NPs and remove them from the circulation within seconds to minutes through the reticuloendothelial system (RES). Imparting a stealth shielding on the surface of these drug delivery systems prevents plasma proteins from recognizing these particles and increasing the systemic circulation time from minutes to hours or even days [17]. Among the several strategies to impart particles with stealth shielding, including surface modification with polysaccharides, poly(acrylamide), and poly(vinyl alcohol), surface modification with PEG proved to be most effective, fueling its widespread use [17-19]. PEG modification is often referred to as PEGylation, and it can prolong exposure of tumor cells to antitumor drug, EPR effect [20], and subsequently increase the therapeutic effect of antitumor drug. PEG offers the advantage that it is non-toxic and non-immunogenic, leading to approved by the FDA for internal use in humans and inclusion in the list of inactive ingredients for oral and parenteral applications.

While it has been demonstrated that PEGylation of NPs causes a greater accumulation of drug at the tumor site by passive targeting, active targeting of the NPs can aid in selection of the target cell type within the tumor site and internalization of the NPs to a greater extent inside the target cells. A wide variety of tumor targeting ligands exist all coupled to nanocarriers. Folic acid (FA) targeting is an interesting approach for cancer therapy [21,22] because it offers several advantages over the use of monoclonal antibodies. More importantly, elevated levels of folate receptors are expressed on epithelial tumors of various organs such as colon, lung, prostate, ovaries, mammary glands, and brain [23,24]. FA is known to be non-immunogenic, and FA-conjugated drugs or NPs are rapidly internalized via receptor-mediated endocytosis. Furthermore, the use of FA as a targeting moiety is believed to bypass cancer cell multidrug efflux pumps [25]. Nevertheless, few literatures reported that both FA and mPEG were loaded onto one kind of chitosan NPs simultaneously.

**Figure 1:**
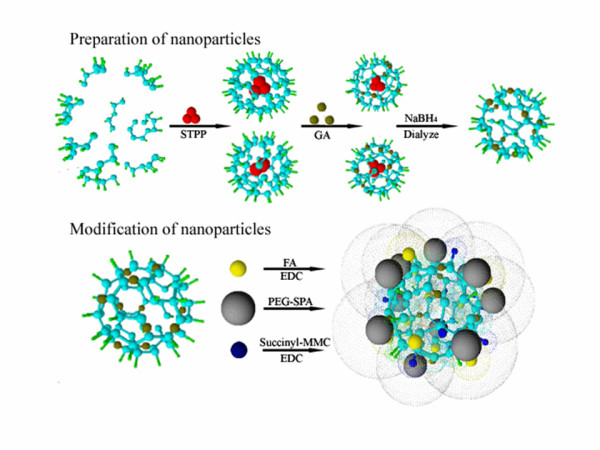
**Schematic illustration of the preparation of NPs and the modification of NPs**. GA, glutaraldehyde; EDC, 1-ethyl-3-(3-dimethylaminopropyl) carbodiimide hydrochloride; STPP, sodium triphosphate.

In this paper, we aim at conjugating both FA and mPEG to the surface of chitosan NPs in order to reach their target, prolong blood circulation, and reduce phagocytosis. Either FA- or mPEG-modified chitosan NPs (FA-NPs or mPEG-NPs) were also prepared for comparison. We chose mitomycin C (MMC) as model drugs to prepare drug-loaded chitosan NPs (MMC-mPEG-FA-NPs) through a covalent coupling. The preparation of NPs and the modification of NPs are illustrated in Figure 1.

## Experimental

### Materials

Chitosan with molecular weight 70,000 (95% degree of deacetylation) was obtained from Zhejiang Aoxing (China). Twenty-five percent glutaraldehyde solution, sodium borohydride, 1-ethyl-3-(3-dimethylaminopropyl) carbodiimide hydrochloride (EDC), and STPP were acquired from Sinoparm Chemical Reagent. FA was purchased from BBI and mitomycin C was purchased from Zhejiang Hisun (China). The 2,000-Da succinimidyl ester of methoxypolyethylene glycol propionic acid (SPA-mPEG) was purchased from Jiaxing Biomatrik (China).

### Preparation of chitosan NPs

One hundred twenty-five-milligram chitosan was dissolved in 100 ml of 1.2% acetic acid and then adjusted the pH to the designated value (pH 5.0) with sodium hydroxide solution (1 M); 16.7 ml of STPP solution (2.5 mg/ml) was slowly added to the chitosan solution under intensive stirring.; then 10 ml of aqueous glutaraldehyde (25%, vol/vol) was added to the resultant mixture, followed by stirring for 12 h at 37°C to form chemical cross-linking NPs, which were isolated by centrifugation at 12,000 rpm for 30 min; and the deposits (NPs) were then resuspended in water, followed by the addition of excessive NaBH_4 _to reduce the C=N bond of NPs. The NPs were isolated by centrifugation again, then the supernatant was decanted, and the NPs were dispersed in 1 M HCl for 12 h and then dialyzed against the distilled water until the pH near 7.0 in order to remove the excess NaBH_4_, STPP, and glutaraldehyde.

### Preparations and characterizations of FA-NPs, mPEG-NPs, and mPEG-FA-NPs

Two milligrams of FA and 4 ml of NPs suspension (5 mg/ml, distilled water used as solvent) were co-mixed in the presence of 10 mg of EDC as catalyst and stirred at room temperature under dark condition for 1 h, and a yellow FA-NPs suspension was obtained. The FA-NPs were collected by centrifugation at 12,000 rpm for 30 min, and the deposit was washed with distilled water and centrifuged again to remove excess FA.

Twenty milligrams of mPEG-SPA and 2 ml of NPs or FA-NP suspensions (10 mg/ml, solvent was distilled water ) were co-mixed and stirred at room temperature under dark condition for 4 h, and then mPEG-NP or mPEG-FA-NP suspensions were obtained. Both mPEG-NP and mPEG-FA-NP suspensions were dialyzed against distilled water to remove the free mPEG-SPA. Fourier transform infrared spectroscopy (FTIR) spectra of different kinds of NPs as well as FA and mPEG-SPA were recorded with KBr pellets on a Nicolet AVATR 360 spectrometer (Nicolet Company) at room temperature, and the spectrums were calculated from 4,000 to 750 cm^-1 ^at 4 cm^-1 ^spectral resolution. The average size, the size distribution, and the zeta-potential of all NPs were measured using Zetasizer Nano ZS (Malvern Instruments). Prior to analysis, 10 ml of distilled water was added to a 20-ml vial containing about 10 mg of each kind of samples.

### Preparation of rhodamine B-labeled NPs

**Figure 2:**
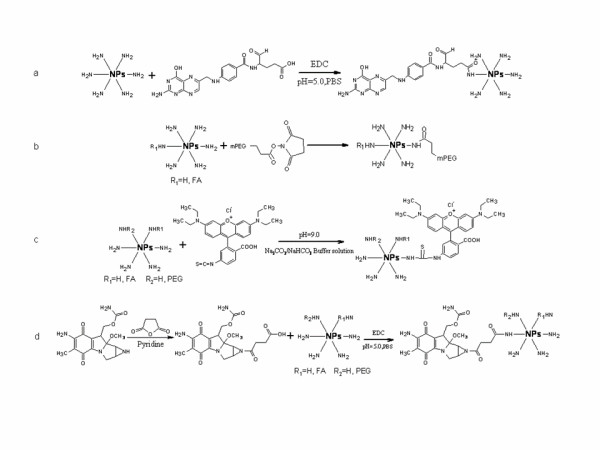
**The reactions involved in this paper**. (**a**) The modification of FA. (**b**) The modification of mPEG. (**c**) Labeled with rhodamine B. (**d**) MMC loaded to the NPs.

Five milligrams of rhodamine B isothiocyanate was dissolved in 1 ml of DMSO. Two hundred microliters of this rhodamine B solution was added to 1 ml of 10 mg/ml different kinds of modified NPs (NPs, FA-NPs, mPEG-NPs, and mPEG-FA-NPs), respectively, and then 1 ml of 2 M pH 9.0 Na_2_CO_3_/NaHCO_3 _buffer was added to the mixtures, which was kept for 12 h at 4°C under dark condition, and the mixture was dialyzed against distilled water to remove the free rhodamine B (Figure 2c).

### Preparation of MMC-mPEG-FA-NPs

Twenty milligrams of MMC and 10 mg of succinic anhydride (molar ratio of succinate/MMC = 2:3) are co-dissolved in 1 ml of pyridine and followed by gentle agitation at room temperature for 10 h, and then pyridine was removed by a Rotavapor and the residue (succinate-modified MMC) was dissolved in 2 ml of pH 5.0 PBS. Both 0.5 ml of the succinate-modified MMC solution and 25 mg of EDC were added to 2 ml of mPEG-FA-NP suspension (8 mg/ml), stirred at room temperature for 1 h, and finally, MMC-mPEG-FA-NPs were collected by centrifugation at 12,000 rpm for 30 min. The deposit (MMC-mPEG-FA-NPs) was washed with a distilled water to get rid of excess EDC and succinate-modified MMC. Then the suspensions were centrifuged again. Lyophilization of the deposit was performed to obtain dry MMC-mPEG-FA-NPs.

The loading content and the loading efficiency of MMC were calculated using the equations listed below.

$$\text{Loading}\;\text{content (\% ) = }\frac{\text{Weight of drug in the nanoparticles}}{\text{Weight of nanoparticles}}\;\times \;100$$

$$\text{Loading}\;\text{efficiency (\% ) = }\frac{\text{Weight of drug in the nanoparticles}}{\text{Weight of the feeding drug}}\;\times \;100$$

### *In vitro *drug release study

The drug releases were carried out in 1/15 M pH 6.3, pH 7.4, and pH 8.3 at 37°C by a dialysis method, respectively. MMC-mPEG-FA-NPs corresponding to 1.7 mg MMC were suspended in 2.5 ml of PBS, and the suspensions were put into a dialysis bag with 3,500 molecular weight cutoff and then the dialysis bag was immersed into 100 ml phosphate buffer, followed by gentle agitation. Periodically, 2 ml of the release medium was withdrawn, and subsequently, the same volume of fresh PBS was added into the release medium and the samples were analyzed by a UV spectrophotometer at 360 nm.

### *In vitro *cellular uptake of different kinds of NPs

Human cervical carcinoma (HeLa) cell lines were provided by the Shanghai Institutes for Biological Sciences, and the cells were grown in Dulbecco's modified Eagle's medium supplemented with 10% fetal bovine serum at 37°C and 5% CO_2_. Nearly confluent cells in 50-ml tissue culture flask were washed twice with Hanks' balanced salt solutions (HBSS) to remove unattached cells and medium. Then the cells were trypsinized by 0.1% trypsin solution and centrifuged at 1,000 rpm for 3 min. The cell pellet was resuspended in fresh media. Cells (2 ml, 5 × 10^7^/L) were plated on 14-mm glass coverslips and allowed to adhere for 12 h. Subsequently, 200 μl (1 mg/ml) of rhodamine B-labeled different kinds of NPs (including NPs, FA-NPs, mPEG-NPs, and mPEG-FA-NPs) was added to the medium, respectively, and incubated for further 24 h. After incubation, the NPs were removed and the wells were washed with ice-cold PBS. The cells were then harvested by trypsinization and centrifuged at 1,000 rpm for 5 min at 4°C. Finally, the cells were resuspended in 500 μl of PBS and stored on ice until analysis. The fluorescence intensity was measured using confocal laser scanning microscopy.

### *In vivo *optical imaging of different kinds of modified NPs in animals

Animal procedures were in agreement with the guidelines of the Institutional Animal Care and Use Committee. Mouse hepatoma-22 cells were implanted subcutaneously into the right hind leg of 4-week-old male nude mice. Biodistributions and imaging studies were performed when tumors reached 0.2-0.5 cm in average diameter. Fluorescence of different modified NPs in nude mice was obtained using the Maestro EX (CRI) in vivo optical imaging system.

### Cell viability assays

A 3-(4,5-dimethylthiazol-2-yl)-2,5-diphenyltetrazolium bromide (MTT) assay was performed to determine cell viability. HeLa cells were chosen for the cell culture experiments. HeLa cells (4 × 10^4^) were seeded into each well of a 96-well cell culture plate. After 24 h culture at 37°C and 5% CO_2 _atmosphere, the cells were exposed to each sample of MMC-mPEG-FA-NP suspension and free MMC solution at a concentration of 12, 18, and 24 μM for 24 h. In addition, untreated cells incubated in HBSS and cell treated with drug-free mPEG-FA-NPs were used as a negative control to which the viabilities of drug-treated cells were compared.

## Results and discussion

### The preparation and characteristics of differential kinds of NPs

**Figure 3:**
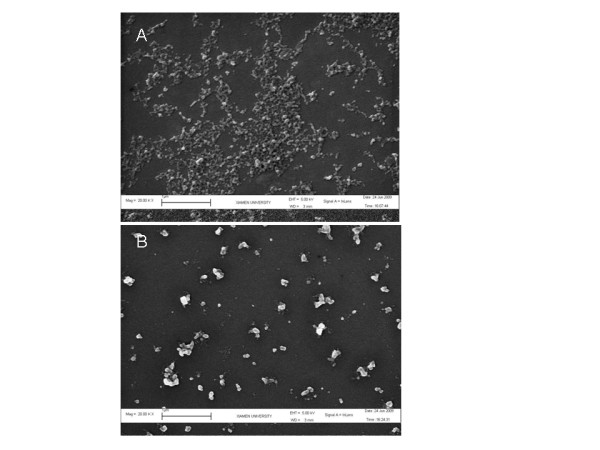
**SME images**. SME image of blank NPs (**A**) and mPEG-FA-NPs (**B**).

Figure 2 shows the process of the preparation and modification of NPs, and the scanning electron microscope image of both blank NPs and mPEG-FA-NPs is shown in Figure 3. Both NPs were essentially spherical in shape, but less cases of the NPs were mono-dispersed particles. It is common that more NPs were in the form of aggregation with each other. Table 1 shows the particles size, size distribution, and zeta-potential of the different kinds of NPs. Both particle size and zeta-potential were the average of triplicate measurements for a single sample. As shown, regardless of all kinds of terminal groups, the chitosan NPs presented a narrow size distribution with an average diameter about 200 nm. The PEGylation reduced the zeta-potential values, confirming the presence of PEG chains shielding the positive charges present at the NP surface. In addition, all modified terminal groups had little influence on particles size, suggesting that the size of NPs was considered to be dominated by the backbone of chitosan, which was related with the molecule weight of chitosan. Nevertheless, detailed influence factors affecting the NP size need to be further approved.

**Table 1:** The average size and the zeta-potential of different kinds of NPs

Sample name	Z-average diameter	Zeta-potential
	
	Mean ± SD (nm)	Mean ± SD (mV)
NPs	202.0 ± 1.4	38.5 ± 0.5
FA-NPs	198.2 ± 1.1	33.1 ± 0.7
mPEG-NPs	209.8 ± 0.8	26.6 ± 0.8
mPEG-FA-NPs	210.4 ± 3.4	28.1 ± 0.4

All of the dates were obtained using the Zetasizer Nano ZS (Malvern Instruments)

**Figure 4:**
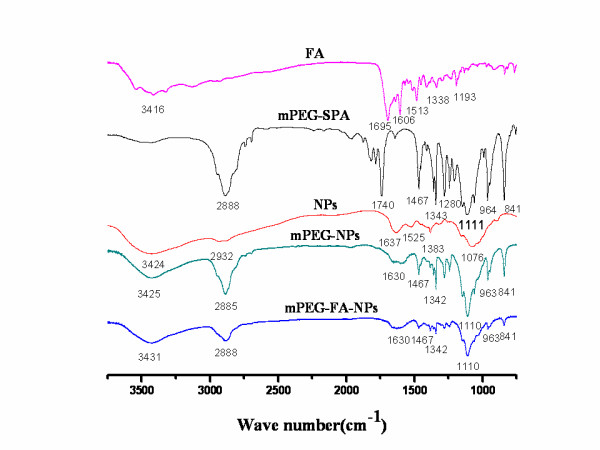
**The FTIR spectroscopy of FA, mPEG-SPA, blank NPs, mPEG-NPs, and mPEG-FA-NPs**.

Figure 4 presents the FTIR spectroscopy of mPEG-SPA, blank NPs, mPEG-NPs, and mPEG-FA-NPs. Characteristic peaks of mPEG-SPA unite are shown in peaks 2,888 and 1,740 cm^-1^. In sample mPEG-FA-NPs, mPEG-SPA peak of 1,740 cm^-1 ^disappeared, but the other characteristic peak of 2,888 cm^-1 ^was observed, indicating that mPEG group was conjugated to chitosan NPs. Typical signals of FA appear at peaks of 1,695 cm^-1^, which also disappeared in sample of mPEG-FA-NPs, together with results of the yellow color of mPEG-FA-NPs obtained, and it was indicated that FA group was also conjugated to chitosan NPs.

### The drug loading efficiency and loading content in NPs

MMC was used as a chemotherapeutic agent by virtue of its antitumor activity. But MMC shows no functional group that could be directly reacted with the NPs. In this study, succinate was chosen as a linker. MMC was reacted with succinic anhydride in advance, and then the succinate-modified MMC (suc-MMC) reacted with mPEG-FA-NPs in the presence of EDC. The loading efficiency and loading content of MMC on mPEG-FA-NPs were 29.2 ± 3.2% and 9.1 ± 1.6%, respectively. The drug-loading content was influenced by the functional group because part of the amino group on the backbone of NPs were consumed by the modifications of mPEG or/and FA and MMC was coupled to NPs through the amino group on the surface of NPs, so the mPEG-FA-NPs had a low drug loading efficiency.

### *In vitro *drug release study

**Figure 5:**
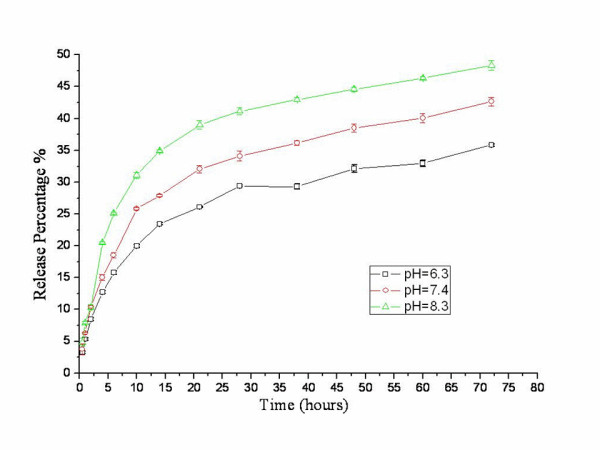
**Drug release from MMC-mPEG-FA-NPs in 1/15 M phosphate buffer at 37°C**. pH = 6.3 (square), pH = 7.4 (circle), pH = 8.3 (triangle).

Figure 5 shows the drug release behaviors of the MMC-mPEG-FA-NPs in pH 6.3, pH 7.4, and pH 8.3 PBS at 37°C, respectively. As the result showed, the drug releases were somewhat biphasic with an initial burst release, followed by a subsequent slower release. The initial burst release should be owed to the presence of free drug absorbed on the surface of NPs, while the sustained drug release was attributed to the cleavage of the chemical bond between MMC and suc-chitosan particles. In addition, longer PEG chain may decrease the cleavage rate of MMC from the chitosan nanoparticles. It is also worth noting that the release profiles show a pH dependence. The higher the medium pH, the faster the release of MMC from the NPs. This is because higher pH weakens the drug-suc-chitosan interaction by deprotonation of the carboxyls in succinate. Since MMC was one of the typical time-dependent drugs, the MMC-mPEG-FA-NPs show an adequate prolonged drug release, suggesting that they have potential as a long-lasting and effective MMC delivery system.

### *In vitro *cellular uptake of different kinds of NPs

**Figure 6:**
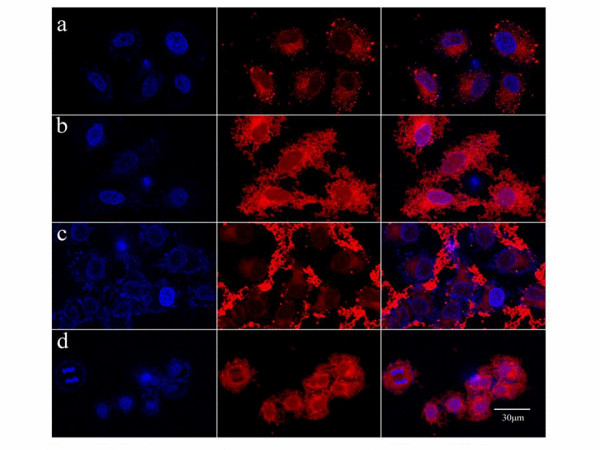
**Confocal images**. Confocal images of HeLa cells after incubated 24 h with different kinds of modified NPs at the same concentration 0.1 mg/ml; the nuclei were stained by DAPI (blue), and all of the NPs are labeled by rhodamine B (red). (**a**) Incubated with pure NPs; (**b**) incubated with FA-NPs; (**c**) incubated with mPEG-NPs; (**d**) incubated with mPEG-FA-NPs.

To visualize the effect of FA-mediated endocytosis of different kinds of modified NPs, the distribution of rhodamine B-labeled NPs on HeLa cells was observed by confocal laser scanning microscopy (Figure 6). By 24-h incubation time, both FA-NPs and mPEG-FA-NPs show high intracellular rhodamine B concentration, which was visualized by red intensity of rhodamine B. Nevertheless, in case of mPEG-NPs, rhodamine B was significantly localized probably in the outside of the cells instead of a distribution in the endosomes, indicating that the mPEG-NPs tended to reduce the cell uptakes. In contrast, for blank NPs (Figure 6a), only low red intensity appeared in the peripheral region of the cells due to slow diffusion process into the cells for 24-h incubation period. NPs are generally internalized into cells via fluid phase endocytosis [26], phagocytosis [27], or receptor-mediated endocytosis [28]. Physicochemical characteristics such as particle size and surface properties played key roles in the cellular of NPs [29]. NP uptake could be considered as an adhesion process followed by an internalization process [30]. However, surface modification of NPs with PEG in our result seems to oppose uptake by the HeLa cells, which is mainly due to the formation of a dense, hydrophilic cloud of long flexible chains on the surface of the NPs that reduces the hydrophobic interactions with the membrane of tumor cells. The chemically anchored PEG chains can undergo spatial conformations, thus preventing the internalization of NPs by the cells. Yet, FA-mPEG-NPs still presented obvious cellular uptake similar to FA-NPs, indicating that PEG modification had minor influence on the FA receptor-mediated intracellular delivery process.

### *In vivo *imaging of different kinds of NPs in animals

**Figure 7:**
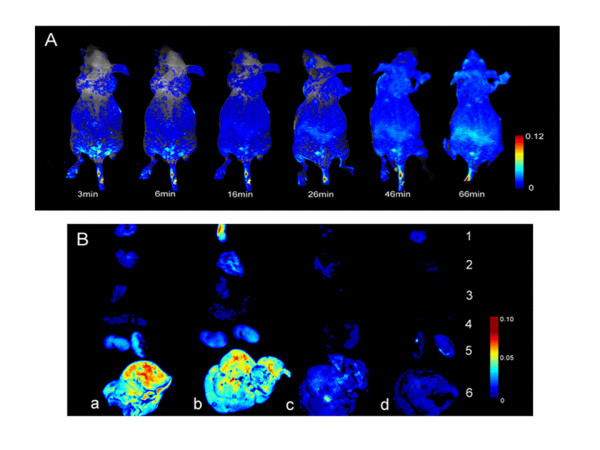
**Fluorescence images**. (**A**) Real-time in vivo fluorescence imaging of intravenously inject with 1 mg NPs without modified at different time points, after injection. (**B**) Representative fluorescence images of dissected organs of nude mice-bearing hepatoma-22 sacrificed 12 h after intravenous injection of different NPs. (a) NPs, (b) FA-NPs, (c) mPEG-NPs, (d) mPEG-FA-NPs. 1, tumor; 2, lung; 3, heart; 4, spleen; 5, kidney; 6, liver. All images were acquired under the same conditions (1 mg NPs per mouse).

Same as the cell tests, all kinds of the NPs were labeled by rhodamine B in advance; 0.2 ml suspension of blank NPs at the concentration of 5 mg/ml was administrated by injection into the tail vein of nude mice, and the resulting images were shown in Figure 7A. Immediately after tail vein injection, fluorescence emitted from the nude was easily visualized in the superficial vasculature of the whole body. Subsequently, as blood circulated, more NPs were deposited in liver.

Considering that biodistribution in tumor-bearing animals may be different from that in normal animals due to some physiological changes brought about by tumor development, hepatoma-22-bearing nudes were employed in the biodistribution investigation. To investigate the distribution of four kinds of NPs in various organs, the nudes were sacrificed immediately after 12 h of intravenous injection, and the amount of NPs within the organs were analyzed by in vivo imaging system to visualize the disposition of the NPs. As shown in Figure 7B, the mPEG-NPs exhibited weakest fluorescence in tumors among the four kinds of NPs, indicating that the mPEG-NPs did not have the ability to specifically bind to tumor. The result also shows that both FA-NPs and mPEG-FA-NPs (Figure 7B b, d) were more fluorescent than NPs without FA-modified (Figure 7B, a, c), suggesting that the accumulations of both FA-NPs and mPEG-FA-NPs in tumors were mediated by folate receptor. This is in agreement with the results of in vitro cellular uptake. In addition, the fluorescence intensity of mPEG-modified NPs (concluding mPEG-NPs and mPEG-FA-NPs) in liver and spleen was significantly lower than that of other kinds of NPs. This observation is consistent with what has been reported in studies with a variety of PEGylated drug delivery systems [31-34]. As reported, mPEGylation can dramatically reduce serum protein adsorption, prevent the attraction of opsonins, and avoid uptake by RES, so as to prolong their residence time in blood and further accumulate in tumor owing to EPR effect.

### Cell viability assays of MMC loaded NPs

**Figure 8:**
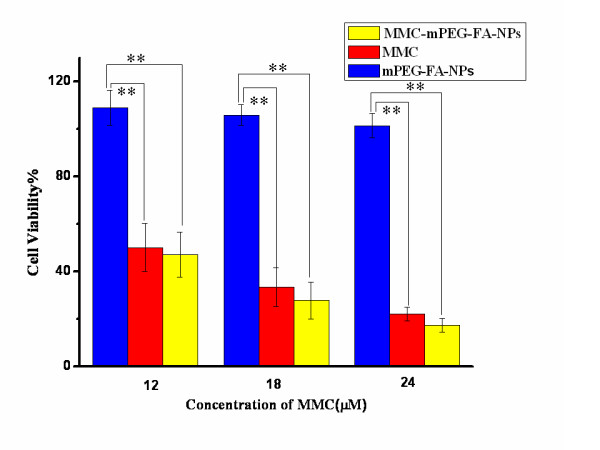
**In vitro viability of HeLa cells**. In vitro viability of HeLa cells treated with a different concentration of free MMC and MMC loaded mPEG-FA-NPs after 24 h. Indicated values were mean ± SD (*n *= 3). **P < 0.01.

The cytotoxic activities of free MMC, drug-free mPEG-FA-NPs, and MMC-mPEG-FA-NPs were evaluated by MTT assay at different concentrations of MMC using the HeLa cell line. Figure 8 shows that the reduction in cell viability by free MMC and MMC-mPEG-FA-NPs was not significantly different, and cell viability was totally suppressed in a concentration-dependent manner after 24 h of incubation. No cytotoxic activity was observed for the drug-free mPEG-FA-NPs, indicating that mPEG-FA-NPs did not affect the mechanism of action of MMC.

It should be emphasized that in the case of MMC-mPEG-FA-NPs, the cytotoxicity observed was only attributed to MMC (dug-free NPs were non-cytotoxic). During the first 24 h of incubation, the significant amount of free MMC released from the nanoparticles could be available to mediate some cytotoxicity. Nevertheless, the cytotoxic effect may be a result of the presence of free MMC or MMC-loaded NPs or a combination of both.

## Conclusion

This novel method to prepare the chitosan NPs was advantageous in terms of a narrow and controllable size distribution. The mPEG-FA-NPs were shown to be taken up by target cells at higher levels than mPEG-NPs and NPs alone. This confirmed that FA retained its targeting ability after conjugation onto NPs and that mPEG-FA-NPs can effectively target the cells overexpressing FA receptors. By combining the biocompatibility and dispersivity of PEG with the specific cell targeting capability of FA, we take advantage of a synergistic effect that results in greatly increased nanoparticle uptake by tumor cells and prolonged blood circulatory time due to reducing the clearance of NPs by the reticuloendothelial system. These results suggest that the synthesized mPEG-FA-NPs can be used as a potentially prolonged anticancer drug carrier for tumor cell-selective targeting treatments.

## Competing interests

The authors declare that they have no competing interests.

## Authors' contributions

Hou ZQ, Yi YF, and Zhang QQ conceived of the study and participated in the design of the study and performed the statistical analysis and drafted the manuscript. Zhan CM and Jiang QW carried out the NPs preparation and its modification studies. Hu Q and Li L carried out the FTIR assays of different kinds of NPs. Chang D and Yang XR participated in the drug release study. Wang YX participated in the study of cellular uptakes of different kinds of NPs in vitro. Ye SF participated in the study of Cell viability assays of MMC loaded NPs, and Xie LY carried out the study of in vivo images in animals and participated in its design and coordination. All authors read and approved the final manuscript.

## References

[B1] Matsumura Y Maeda H A new concept for macromolecular therapeutics in cancer chemotherapy: mechanism of tumoritropic accumulation of proteins and the antitumor agent smancs Cancer Res 1986 46 6387 2946403

[B2] Duncan R Polymer conjugates for tumour targeting and intracytoplasmic delivery: the EPR effect as a common gateway Pharm Sci Technol Today 1999 2 441 10.1016/S1461-5347(99)00211-4 10542390

[B3] Lee KY Kim JH Kwon IC Jeong SY Self-aggregates of deoxycholic acid-modified chitosan as a novel carrier of adriamycin Colloid Polym Sci 2000 278 1216 10.1007/s003960000389

[B4] Ruel-Gariepy E Leclair G Hildgen P Gupta A Leroux JC Thermosensitive chitosan-based hydrogel containing liposomes for the delivery of hydrophilic molecules J Control Release 2002 82 373 10.1016/S0168-3659(02)00146-3 12175750

[B5] Hirano S Chitin and chitosan as novel biotechnological materials Polym Int 1999 48 732 10.1002/(SICI)1097-0126(199908)48:8<732::AID-PI211>3.0.CO;2-U

[B6] Molinaro G Leroux J Damas J Adam A Biocompatibility of thermosensitive chitosan-based hydrogels: an in vivo experimental approach to injectable biomaterials Biomaterials 2002 23 2717 10.1016/S0142-9612(02)00004-2 12059021

[B7] Park JH Cho YW Chung H Kwon IC Jeong SY Synthesis and characterization of sugar-bearing chitosan derivatives: aqueous solubility and biodegradability Biomacromolecules 2003 4 1087 10.1021/bm034094r 12857096

[B8] Dufes C Schätzlein AG Tetley L Gray AI Watson DG Olivier JC Couet W Uchegbu IF Niosomes and polymeric chitosan based vesicles bearing transferrin and glucose ligands for drug targeting Pharm Res 2000 17 1250 10.1023/A:1026422915326 11145231

[B9] Hejazi R Amiji M Chitosan-based gastrointestinal delivery systems J Control Release 2003 89 151 10.1016/S0168-3659(03)00126-3 12711440

[B10] Berthold A Cremer K Kreuter J Preparation and characterization of chitosan microspheres as drug carrier for prednisolone sodium phosphate as model for antiinflammatory drugs J Control Release 1996 39 17 10.1016/0168-3659(95)00129-8

[B11] Tian XX Groves MJ Formulation and biological activity of antineoplastic proteoglycans derived from mycobacterium vaccae in chitosan nanoparticles J Pharm Pharmacol 1999 51 151 10217313 10.1211/0022357991772268

[B12] Ohya Y Shiratani M Kobayashi H Ouchi T Release behavior of 5-fluorouracil from chitosan-gel nanospheres immobilizing 5-fluorouracil coated with polysaccharides and their cell specific cytotoxicity J Macromol Sci Pure Appl Chem 1994 31 629

[B13] Bodmeier R Oh KH Pramar Y Preparation and evaluation of drug-containing chitosan beads Drug Dev Ind Pharm 1987 15 1475

[B14] Tokumitsu H Ichikawa H Fukumori Y Chitosan-gadopentetic acid complex nanoparticles for gadolinium neutron-capture therapy of cancer: preparation by emulsion-droplet coalescence technique and characterization Iran J Pharmaceut Res 1999 16 1830 10.1023/a:1018995124527 10644070

[B15] Thanoo BC Sunny MC Jayakrishnan A Cross-linked chitosan microspheres: Preparation and evaluation as a matrix for controlled release of pharmaceuticals J Pharm Pharmcol 1992 44 283 10.1111/j.2042-7158.1992.tb03607.x 1355537

[B16] Gupta KC Jabrail FH Controlled-release formulations for hydroxy urea and rifampicin using polyphosphate-anion-crosslinked chitosan microspheres J Appl Polym Sci 2007 104 1942 10.1002/app.25881

[B17] Owens DE Peppas NA Opsonization, biodistribution, and pharmacokinetics of polymeric nanoparticles Int J Pharm 2006 307 93 10.1016/j.ijpharm.2005.10.010 16303268

[B18] Veronese FM Pasut G PEGylation, successful approach to drug delivery Drug Discov Today 2005 10 1451 10.1016/S1359-6446(05)03575-0 16243265

[B19] Park JH Lee S Kim JH Park K Kim K Kwon IC Polymeric nanomedicine for cancer therapy Prog Polym Sci 2008 33 113 10.1016/j.progpolymsci.2007.09.003

[B20] Sheng Y Liu CS Yuan Y Tao XY Yang F Shan XQ Zhou HJ Xu F Long-circulating polymeric nanoparticles bearing a combinatorial coating of PEG and water-soluble chitosan Biomaterials 2009 30 2340 10.1016/j.biomaterials.2008.12.070 19150737

[B21] Sun C Sze R Zhang M Folic acid-PEG conjugated superparamagnetic nanoparticles for targeted cellular uptake and detection by MRI J Biomed Mater Res 2006 78 550 10.1002/jbm.a.30781 16736484

[B22] Kim SH Jeong JH Chun KW Park TG Target-specific cellular uptake of PLGA nanoparticles coated with poly(L-lysine)-poly(ethylene glycol)-folate conjugate Langmuir 2005 21 8852 10.1021/la0502084 16142970

[B23] Sudimack J Lee RJ Drug targeting via the folate receptor Adv Drug Deliv Rev 2000 41 147 10.1016/S0169-409X(99)00062-9 10699311

[B24] Goren D Horowitz AT Tzemach D Tarshish M Zalipsky S Gabizon A Nuclear delivery of doxorubicin via folate-targeted liposomes with bypass of multidrug-resistance efflux pump Clin Cancer Res 2000 6 1949 10815920

[B25] Leamon CP Reddy JA Folate-targeted chemotherapy Adv Drug Delivery Rev 2004 56 1127 10.1016/j.addr.2004.01.008 15094211

[B26] Shoepf U Marecos E Melder R Jain R Weissleder R Intracellular magnetic labeling of lymphocytes for in vivo trafficking studies Bio Techniques 1998 24 642 10.2144/98244rr01 9564539

[B27] Weissleder R Cheng HC Bogdanova A Magnetically labeled cells can be detected by MR imaging J Magn Reson Imaging 1997 7 258 10.1002/jmri.1880070140 9039625

[B28] Lee R Kim PH Choi JW Oh-Joon K Kim K Kim D Yun CO Yoo KH Capacitance-based real time monitoring of receptor-mediated endocytosis Biosensors and Bioelectronics 2010 25 1325 10.1016/j.bios.2009.10.025 19932608

[B29] Alexis F Pridgen E Molnar LK Farokhzad OC Factors affecting the clearance and biodistribution of polymeric nanoparticles Mol Pharm 2008 5 505 10.1021/mp800051m 18672949 PMC2663893

[B30] Gao H Shi W Freund LB Mechanics of receptor-mediated endocytosis Proc Natl Acad Sci USA 2005 102 9469 10.1073/pnas.0503879102 15972807 PMC1172266

[B31] Park JH Lee S Kim JH Park K Kim K Kwon IC Polymeric nanomedicine for cancer therapy Pro Polym Sci 2008 33 113 10.1016/j.progpolymsci.2007.09.003

[B32] Shi B Fang C Pei YY Stealth PEG-PHDCA niosomes: effects of chain length of PEG and particle size on niosome surface properties, in vitro drug release, phagocytic uptake, in vivo pharmacokinetics and antitumor activity J Pharm Sci 2006 95 1873 10.1002/jps.20491 16795003

[B33] Jiang YY Tang GT Hong MH Zhu SJ Fang C Shi B Pei YY Active tumor-targeted delivery of PEG-protein via transferrin-transferrin-receptor system J Drug Target 2007 15 672 10.1080/10611860701603414 18041635

[B34] Pirollo KF Chang EH Does a targeting ligand influence nanoparticle tumor localization or uptake? Trends Biotechnol 2008 26 552 10.1016/j.tibtech.2008.06.007 18722682

